# The explanatory power of social cognitive theory in determining knowledge sharing among Saudi faculty

**DOI:** 10.1371/journal.pone.0248275

**Published:** 2021-03-19

**Authors:** Abdullah Almuqrin, Ibrahim Mutambik

**Affiliations:** Department of Information Science, King Saud University, Riyadh, Saudi Arabia; University of Macau, MACAO

## Abstract

Knowledge sharing positively and significantly improves academics’ research, instruction and community service. The study of knowledge sharing in Saudi higher education is limited and offers little guidance to administrators, as well as faculty on how to capitalize on knowledge sharing and utilize it to their own and organizations’ benefits. This research presents findings from an original quantitative study testing a structural equation model linking social cognitive theory to knowledge sharing collection and donation measured by validated scales from the extant literature. Community characteristics including altruism and reputation carry significant positive effects on knowledge sharing collection and donation. Similarly, personal outcomes expectations possess a moderate positive effect on knowledge sharing collection and donation. Self-efficacy in knowledge sharing emerged as an important predictor of knowledge sharing activities among Saudi academics. The findings suggest the need for developing professional training seminars on using social media for knowledge sharing in formal departmental and college settings. Further, the results confirm the relevance of social cognitive theory for the study of knowledge sharing. This creates the need for Saudi universities to invest in mentorship programs using digital platforms where personal and community outcomes’ expectations are likely to improve among academics thereby increasing knowledge sharing activity.

## Introduction

Knowledge Sharing (KS) improves faculty’s domain-specific knowledge, skills, and abilities [1]. It increases instructional effectiveness through sharing effective teaching materials among colleagues and peers [2]. It increases research productivity through sharing tacit specialized knowledge on research methodologies, techniques, funding sources and field-specific norms unpublicized in writing [3]. Knowledge sharing improves organizational work by sharing experiences among departments and offices that leads to the improved provision services to students and internal stakeholders [4]. The plethora of benefits of knowledge sharing on higher education institutions warrants up-to-date research on the determinants of knowledge sharing among faculty especially in previously neglected settings like the Saudi higher education [5].

Many problems face Saudi higher education knowledge sharing activities [6]. The full potential of knowledge sharing in Saudi higher education is not realized causing a significant decrease in the effectiveness and efficacy of the sector in delivering its educational, community and research services [7]. Alsuraihi, Yaghi and Nassuora [8] reported that Saudi faculty have a weak rate of sharing teaching materials, high absenteeism for departmental, college or university administrative meetings and low levels of interdisciplinary collaborations. Saudi higher education institutions suffer from low knowledge sharing rates due to diminished explicit knowledge infrastructure and reliance on informal techniques for tacit knowledge circulation. Alhazmi [7] concluded that Saudi faculty heavily utilize verbal techniques to share knowledge on instruction, research, or service. Many universities and colleges do not have extensive written explicit knowledge guiding the work of faculty or staff. Tacit knowledge sharing is an essential process often accomplished using oral means of communication in Saudi higher education [8].

The problem addressed in this manuscript is the lack of sufficient knowledge sharing among Saudi faculty resulting in diminished work productivity, institutional performance, and quality [6–8]. A related managerial challenge is the decreased effectiveness and efficiency resulting from the over reliance on verbal techniques to share knowledge, and the absence of formal organizational infrastructure to streamline implicit and explicit knowledge donation, collection and dissemination among personnel and various units within higher learning institutions [9, 10]. To remedy this deficiency, this research investigates the effectiveness of social cognitive theory to aid in the improvement of KS activities in Saudi higher education [11, 12]. To fulfill this end, the following research questions were proposed, and investigated: 1) to what extent does knowledge sharing self-efficacy among Saudi faculty influence their KS donation and collection? 2) To what extent does personal expectations from KS activities drive Saudi faculty’s donation and collection of knowledge in their work environments? 3) To what extent does community expectations about KS activities change Saudi faculty’s KS donation and collection endeavors?

The motivation underlying this research endeavor is the improvement of knowledge sharing at the individual faculty level, as well as at the institutional level within the Saudi higher education system. The need for conducting the contemporary analysis lies in the design of appropriate interventions that are theoretically grounded to enhance knowledge sharing collection, organization, and dissemination on colleges and universities campuses in Saudi Arabia. New faculty members comprise the largest share of the instructional staff in Saudi universities, and such a workforce is unprepared for undertaking major research, teaching or funding endeavors, which make knowledge sharing exceptionally important in this context. The benefits of the current analysis are plentiful. First, interventions at all levels and scopes could be designed using the findings of the analysis to improve individual and organizational efforts of knowledge sharing amelioration. Second, policymakers and administrators are able to target areas of deficiencies to fulfill faculty’s needs and increase their KS activities. Third, individual faculty learn how to distribute the knowledge effectively and efficiently they aspire to share with their peers or work units.

Using responses from 411 faculty at a large Saudi university and structural equation modelling performed on AMOS 22, a total of 12 hypotheses were supported indicating not only the validity and reliability of the 7 scales comprising the questionnaire, but also the relevance of social cognitive theory in explaining knowledge sharing activities. Faculty’s personal expectations and community expectations were statistically significant in predicting Saudi faculty’s knowledge sharing collection and donation activities. More importantly, self-efficacy in knowledge sharing emerged as one of the most important predictors of knowledge sharing activities. Similarly, altruism was the strongest predictor of knowledge sharing demonstrating the importance of culture when investigating knowledge sharing in any setting including higher education.

## Literature review

### Conceptualizing knowledge sharing in higher education

Knowledge sharing is the systematic collection and dissemination of tacit and explicit information with others for the purposes of self-development, increasing work-related effectiveness and efficiency and decreasing waste and maximizing productivity for a given task or job [1]. Knowledge sharing in higher education is a comprehensive phenomenon that covers both the exchange, as well as transfer of knowledge. The former relates to the voluntary donation of knowledge among peers, departments and colleges while the latter pertains to the movement of knowledge from one unit to the other in a more formal setting [2]. This conceptualization includes unidirectional, bidirectional, and multidirectional knowledge exchange, transfer and sharing across any set of individuals, units or institutions in higher education [13]. Knowledge sharing takes a variety of forms in higher education including formal and informal settings. The former relates to scheduled meetings, conferences, conventions, or any planned activity aimed at knowledge organization, collection, exchange, or dissemination. Informal settings include private discussions among peers, one-to-one assistance sessions among colleagues, ad-hoc requests for help with a given task by a peer or director/chair/dean, unplanned luncheons and the like [3]. Notice that all formal and informal events could take place virtually, as well as in physical environments.

### Limitations of knowledge sharing initiatives in higher education

Extensive research on the challenges of knowledge sharing faces in higher education has noted the presence of organizational, technological, and individual level factors hampering higher education institutions from reaping the full potential of knowledge sharing [14]. First, the absence of a shared vision by university offices, colleges and departments on the role of knowledge sharing, and how it should be utilized in advancing the mission of institutions thwart knowledge sharing efforts and dilute their impact on the organization, as well as its individuals [15]. Further, the lack of shared physical and virtual digital platforms where explicit and implicit knowledge are located for anyone in the organization to access and use present another challenge for knowledge sharing implementation [4]. Departments, research units, groups and colleges all have differing roles and objectives leading to a high level of idiosyncrasy, which is poorly documented, translated into explicit knowledge and made available to everyone in the organization for immediate utilization [16]. In addition, academic’s lack of trust or perceived loss of prestige once knowledge is shared with others prevent many from transferring or exchanging explicit and implicit knowledge. The literature has concluded that higher education institutions have failed to materialize efforts of knowledge sharing that overcome such barriers.

### Knowledge sharing in Saudi higher education

The limited empirical research on Saudi faculty’s knowledge sharing activities concluded that academics in Saudi Arabia prefer oral methods of communicating explicit and tacit knowledge [17]. Alsuraihi, Yaghi and Nassuora [8] found that approximately 96% of the 210 faculty surveyed at a major public research institution in Jeddah preferred the oral method of communicating their research to their peers. Similarly, Alsaadi [6] concluded that a large proportion of the 140 faculty participants chose non-written formal communication mediums to deliver important information to their peers on their work, or the university’s policies and practices.

In line with the non-institutional sanctioned infrastructure for KS activities, research on Saudi faculty’s KS practices confirmed a high likelihood of utilizing non-work-related mediums like social media platforms to share work-related knowledge [6, 7, 18]. One study found that 81% of a 210 faculty sample surveyed at a large university, reported the use of Facebook and Twitter to share implicit and explicit knowledge with their peers, department chairs and college deans [8]. Alsaadi [6] concluded that a large proportion of Saudi faculty utilize their personal social media accounts to communicate information to their peers, students and organizations with respect to their research, specialized skills sets, and peculiar organizational knowledge. In the same vein, Alhamadi [18] noted that Saudi doctoral students in the United States who are on their way of becoming tenured faculty upon the completion of their programs heavily utilize their personal accounts on social media to share all types of research, funding, writing, and compliance knowledge.

Previous empirical investigations on Saudi faculty practices of KS yielded a low rate of donation and collection of implicit and explicit knowledge for the advancement of departments, colleges, institutions, and the overall higher education system [6, 8, 10]. In a cross-sectional survey-based study [8] found that KS activities were largely driven by faculty’s desire to develop in their careers and improve their personal research skills. [10] reported that Saudi faculty are not interested in the enhancement of their departments or colleges, and are heavily motivated by the desire to be promoted in their careers via publishing more peer-reviewed journals, which explain the high rate of knowledge sharing activities devoted to publications. Alsaadi [6] highlighted the low participation rate of Saudi faculty in administrative, and organizational knowledge sharing activities that are likely to improve the infrastructure of KS at their institutions.

Research on KS in Saudi higher education notes to the pressing need of institutions to build electronic mediums of KS infrastructure [5, 9, 10]. Ghabban, Selamat and Ibrahim [9] noted the low number of institutional channels on their websites, social media platforms or other electronic forms of KS despite the strong preference for faculty to utilize computer-mediated KS methods. By the same token, Shafique [10] pointed to the important role of digital libraries as information centers that could facilitate knowledge sharing in Saudi higher education, which are underdeveloped across many Saudi colleges and universities [5] recommended the installation of a publicly free national database to communicate implicit and explicit knowledge related to faculty research, teaching and service by a consortium comprised of all higher learning institutions in Saudi Arabia.

The empirical study of KS in Saudi higher education points to the absence of organizational rewards to faculty engaging in KS activities [19]. Alsaadi [6] reported low levels of top management support for KS activities, and a high level of mistrust among members of the same institution preventing many from donating knowledge. Commenting on a similar issue, Shafique [10] concluded that Saudi universities and colleges lack clear visions, and implementation strategies for KS. Similarly, Alhazmi [7] found that the lack of managerial recognition for KS activities, and incentives attached to its practice as the most important reasons behind the low practice of KS among Saudi faculty. Furthermore, [20] and [8] concluded that Saudi universities and colleges do not actively pursue KS, which make faculty not prioritize it vis-à-vis other work-related functions.

Research on Saudi faculty knowledge sharing utilization indicated the importance of personal and communal expectations of knowledge sharing among academics in predicting knowledge sharing behaviors [21] reported high perceived future collaboration and assistance associated with KS activities among faculty in Saudi institutions. Similarly, Alammari and Chandran [22] found that Saudi faculty are more likely to engage in KS if it brings them personal career advancement, research development and funding benefits in the future. By the same token, Alammari and Chandran [23] suggested that if Saudi faculty believe that their work environments become more collaborative, interactive and trustworthy, they are more likely to practice KS.

#### Theoretical framework: Social cognitive theory

**Fig 1:**
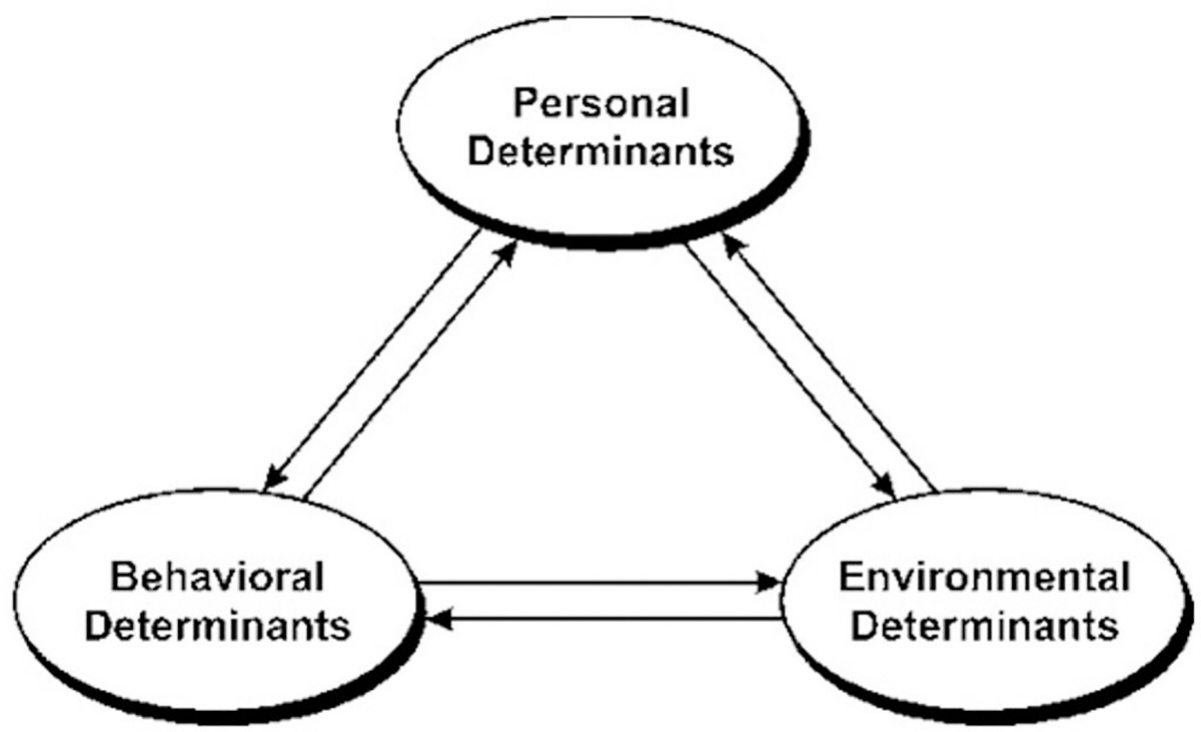
Social cognitive theory basic structure.

Social cognitive theory is a psychological explanation for the initiation and maintenance of human behavior. Bandura [24, 25] posited that personal factors like perceptions, feelings, predispositions, demographic and biological characteristics influence the probability an individual adopts and practices a certain behavior, as well as the environment around him or her. By the same token, environmental factors like the family, networks, work or planned and fortuitous events determine whether an individual partakes in a behavior or not, as well as the individual characteristics of the person. Finally, behaviors themselves influence the environment, as well as the personal factors causing what Bandura referred to as the triadic reciprocal determinism among the three broad domains of factors. This behavioral framework is different from others in the fact that it does not assume a linear modelling approach where a number of specified constructs determine behavior in a clear exogenous-endogenous manner. On the contrary, social cognitive theory explicitly postulates that individual behavior is determined by many personal and environmental factors, and a determinant of them as well. Fig 1 depicts the basic structure of the theoretical framework.

### Social cognitive theory and knowledge sharing

Knowledge sharing like any other behavior is an appropriate phenomenon to be analyzed with the lens of social cognitive theory. Individual’s engagement in knowledge sharing is determined by their personal beliefs, expectations, past experiences, and capabilities concerning knowledge sharing [26]. Their engagement is also determined by whether those around them at work, home or elsewhere are engaging in knowledge sharing, how knowledge sharing is viewed by their immediate circles and the wider public and whether knowledge sharing behaviors yield any social expectations or not [27]. Knowledge sharing engagement is also influenced by the access of individuals to those engaged in knowledge sharing and their knowledge sharing products offering them a fertile ground for observational learning. Individual’s own past knowledge sharing behaviors determine a portion of their present and future knowledge sharing behaviors [28]. All in all, a confluence of cognitive and social factors determines the knowledge sharing behaviors anyone engages in.

Despite few exceptions, empirical research has found consistent support for social cognitive theory’s explanatory power for knowledge sharing behaviors. In their well-cited study on the effects of social cognitive theory on knowledge sharing among Taiwanese virtual community members knowledge sharing behaviors, Chiu, Hsu and Wang [29] found community-related expectations to be a significant predictor of knowledge sharing quality and quantity (path coefficients of 0.28 S and 0.32 respectively). In the country, the study reported non-significant associations between personal expectations and knowledge sharing quality or quantity. The authors conjectured that this unexpected finding may be due to the measurement and sampling biases of their study. In a meta-analysis of 28 studies published between 1994 and 2008 with 117 unique bi-lateral correlations, Liang, Liu and Wu [30] found perceived benefits to be a moderate positive significant predictor of knowledge sharing behaviors, r = 0.37. By the same token, organizational rewards systems predicted knowledge sharing behaviors the best, r = 0.41. The same study reported that the average effect size for organizational support and organizational commitment on knowledge sharing behaviors were r = 0.23 and r = 0.37 respectively. In a meta-analysis exploring the effect size of individual and organizational factors of knowledge sharing behaviors, Kumari and Takahashi [31] reported that knowledge sharing self-efficacy has an average correlation of r = 0.28 pm knowledge sharing behaviors using 7 independent effect sizes covering 1771 participants. Using 14 unique effect sizes on 3973 respondents, the authors reported that perceived expectations/attitude towards knowledge sharing has an average effect size on knowledge sharing behavior of r = 0.46. Subjective norms, the extent to which an individual is influenced by the attitudes of those surrounding him/her, was found to have a positive significant average effect size of r = 0.40.

### Proposed conceptual model

Knowledge sharing as a behavior could be explained from a variety of perspectives. Previous research utilized different behavioral models like the theory of planned behavior, social capital theory and social cognitive theory. The empirical evidence generated from such literatures has been contradictory. One of the most stable findings is that social cognitive theory possesses the best fit when modelled against varying samples, contexts and measurements, and therefore it appears to be the most appropriate theoretical framework from an empirical standpoint. This support is aided by many proposed mechanisms linking personal, and environmental factors with knowledge sharing outlined below.

**Fig 2:**
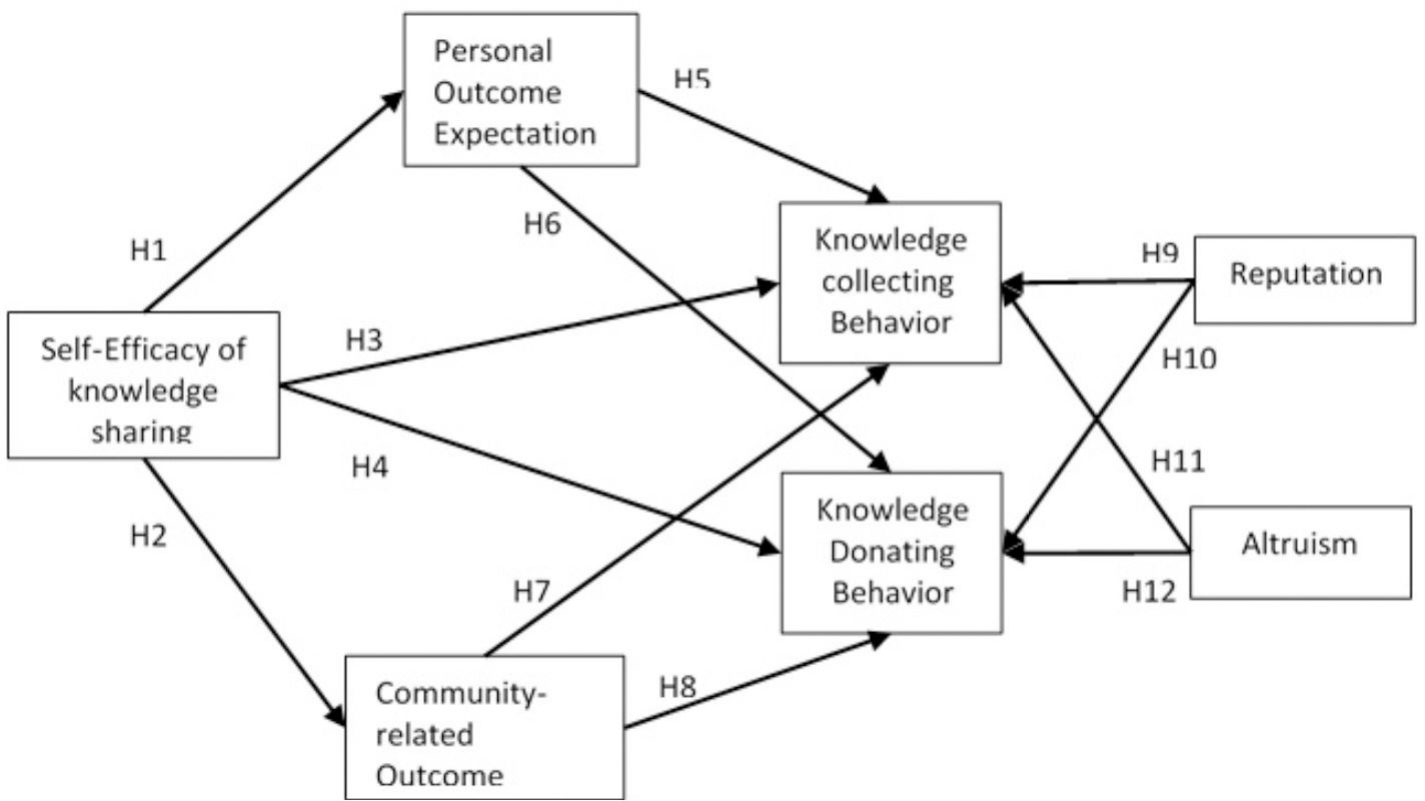
Research model.

Previous research on faculty’s knowledge sharing behaviors noted that their self-efficacy, personal expectations, and their beliefs about how their peers, academic and industry-related communities to their field of study interpret their knowledge sharing practices [11]. This makes the case for considering the three personal factors: self-efficacy, personal expectations, and community expectations as the primary cognitive drivers for knowledge sharing behaviors among faculty [12]. Studies of faculty knowledge sharing behaviors have also highlighted the explanatory power of reputation and altruism on their attitudes and behaviors related to knowledge sharing [32]. The environment of higher education dictates that for a faculty to succeed in his/her career, he/she needs to establish a good reputation in the field of study, unit of work and the community at large to reap all expected benefits and aspirations attached to an academic career [33]. In addition, in higher education environments, collaboration on all kinds of projects, voluntary provision of key information, donating time for non-instructional or teaching duties as the for departments, colleges or offices on campus or even helping a community member solve a problem are all part of an academic career, and all share some elements of altruism [34]. Reputation and altruism are two social determinants of the faculty’s tendency to share knowledge. Fig 2 depicts the conceptual framework using social cognitive theory to predict Saudi faculty’s knowledge sharing behaviors.

### Hypotheses development

#### Self-efficacy and personal outcome expectations

Knowledge sharing behavior, as well as knowledge sharing personal expectations are partly determined by the faculty’s self-efficacy, which refers to the academic confidence in performing knowledge sharing activities despite the presence of cognitive and environmental challenges [35]. Faculty knowledge sharing self-efficacy influences their personal expectations of knowledge sharing [36, 37]. Heightened knowledge sharing self-efficacy makes faculty more likely to demonstrate their skills, abilities, and knowledge through translating their tacit knowledge into documented explicit knowledge through knowledge sharing activities, which gives them a positive feeling about themselves and their confidence in their own fields [38, 39]. Elevated self-efficacy in knowledge sharing among faculty encourages faculty to help others who may be in need of knowing some type of information, which is translated in knowledge sharing activities that give the faculty a good feeling of being an amicable contributor to his/her field, team, department or the community [38]. Knowledge sharing self-efficacy among faculty enhances faculty’s perceptions about their odds of obtaining some benefits linked to their career development since they are likely to share all kinds of knowledge they have with others, who are likely to reciprocate back by giving tangible rewards for the knowledge sharing behaviors performed by the faculty [40]. All such links between self-efficacy and faculty’s personal expectations concerning knowledge sharing give rise to the following hypothesis.

H_1_: Faculty’s knowledge sharing self-efficacy is positively associated with faculty’s knowledge sharing personal outcome expectations.

#### Self-efficacy and community outcome expectations

Knowledge sharing faculty’s community-related expectations are partially explained by faculty’s knowledge sharing self-efficacy [41, 42]. Academics’ knowledge sharing self-efficacy makes them more likely to perform knowledge sharing believing that it enhances their networks’ value [35]. They believe that if they perform knowledge sharing activities, which is a reflection of their self-efficacy levels of knowledge sharing, others in the network are likely to perform knowledge sharing, which totals to more knowledge being shared [35]. In addition, knowledge sharing self-efficacy improves faculty’s perceptions about trust and reciprocity [43], which are two good normative qualities of networks, groups, teams or overall institutions. They believe that if they perform knowledge sharing, which requires high self-efficacy [44], other members in their respected units or networks will be more likely to share knowledge, which strengthens the bond linking members, survival of the network and reach of the group/team [40]. Arising from this logic, the following hypothesis is constructed.

H_2_: Faculty’s knowledge sharing self-efficacy is positively associated with faculty’s knowledge sharing community outcomes expectations.

#### Self-efficacy and knowledge collection

Faculty’s knowledge sharing self-efficacy drives knowledge collection behaviors upwards. Knowledge sharing self-efficacy makes faculty more likely to perform knowledge sharing activities [40]. This increases their practice of knowledge collection [45]. Knowledge sharing self-efficacy equips faculty with more resistance to time and situational constraints that could interfere with their commitment to knowledge collection preparing for knowledge sharing activities [38, 46]. Self-efficacy also awards faculty’s the ability to tolerate errors, mistakes and inefficiencies associated with information collection and documentation [47]. Self-efficacy gives faculty the quality of incremental improvement in data, information and knowledge collection given their high perseverance rate for withstanding all obstacles involved in knowledge sharing activities [38]. The argument purported above gives emergence to the following hypothesis.

H_3_: Faculty’s knowledge sharing self-efficacy is positively associated with faculty’s knowledge collection behaviors.

#### Self-efficacy and knowledge donation

Faculty’ knowledge sharing donation is partly determined by their knowledge sharing self-efficacy [48]. Once faculty feel confident in the quality of their information, and the way to communicate it with others, they are more willing to donate it [35, 49]. In addition, faculty who believe that they are capable of transforming their specialized tacit knowledge into something worthy of donating on a forum, group chat or platform with others, they are more likely to share such knowledge [40]. Faculty that have a record of knowledge sharing and feel confident in their knowledge sharing abilities are more likely to feel that they could be of utmost help to their peers or organizations, and therefore donate more knowledge [50]. Self-efficacy in knowledge sharing gives faculty a sense of obligation to help others to overcome similar obstacles experienced by themselves [35], making them engaged in knowledge sharing more frequently compared to faculty with lower knowledge sharing self-efficacy [35]. The foregoing discussion helps construct the following hypothesis.

H_4_: Faculty’s knowledge sharing self-efficacy is positively associated with faculty’s knowledge donation behaviors.

#### Personal outcomes expectations and knowledge sharing collection

Faculty’s personal expectations of knowledge sharing determine a portion of their knowledge sharing collection behaviors [51]. When faculty believe that knowledge sharing improves their own skills, abilities, and knowledge, they begin to search for useful information, and most importantly collect, as well as organize them in a useful manner for different purposes [52, 53]. Faculty believe this improves their career prospects, and therefore start amassing new information they perceive as useful for professional advancement [52]. Faculty value knowledge sharing for its potential effects on their research productivity, instructional effectiveness and impactful service, and therefore they seek new resources, information and knowledge from various references to formulate a collection of explicit and tacit knowledge [40]. From this discussion, one may propose the following hypothesis.

H_5_: Faculty knowledge sharing personal expectations are positively associated with knowledge sharing collection behaviors among faculty.

#### Personal outcomes expectations and knowledge sharing donation

Faculty’s personal expectations attached to knowledge sharing activities explain some variance in their knowledge sharing donation behaviors [54]. Faculty who expect that they would receive recognition, reputation boost or acclamations from knowledge sharing are more likely to donate knowledge sharing to their peers, as well as communities they are part of [52]. Furthermore, faculty who believe that knowledge sharing activities will make them more marketable within their organizations or fields to assume future leadership positions will likely donate knowledge gesturing their collaborative and cooperative executive skills and styles [52, 55]. In addition, faculty who perceive knowledge sharing as an enhancing activity for their personal development, as well as those involved within their own field are more likely to donate knowledge to contribute to the common good of the team, group, or the field at large [38]. What follows from the current discussion is the hypothesis spelled out below.

H_6_: Knowledge sharing personal expectations are positively associated with knowledge sharing donation behaviors among faculty.

#### Community expectations and knowledge collection

Community-related expectations of knowledge sharing determine a portion in the variance of knowledge sharing collection behaviors among faculty [35, 56]. Faculty who believe that knowledge sharing activities will advance the status and value of his/her networks will be more likely to collect information conducive to the advancement of the organization or collective [35]. On another note, faculty who believe that their communities or groups are important, and their survival is essential to accomplish a specific vision or mission will be more likely to collect explicit and tacit knowledge to benefit the group in any way, shape or form [52]. Faculty who prefer to establish a community bond with peers or co-workers are likely to collect information that serves such a purpose, and eventually share it with others [57]. Therefore, one may hypothesize the following.

H_7_: Knowledge sharing community expectations are positively associated with knowledge sharing collection behaviors among faculty.

#### Community expectation and knowledge donating

Community-related expectations pertaining knowledge sharing explain part of knowledge sharing donation behaviors exhibited by faculty [38]. Faculty who value the norms of reciprocity, collaboration, cooperation and coordination are more likely to donate their tacit and explicit knowledge to advance such normative qualities of groups or teams [38]. In addition, faculty who believe that their field will be advanced or achieve a better status are more likely to donate knowledge to realize such an end [35]. Faculty who believe that knowledge sharing correlates positively with engagement, commitment and loyalty to their disciplines or specific areas of research are more likely to donate knowledge to advance such objectives [58]. This argument makes the reader hypothesize the following.

H_8_: Knowledge sharing community expectations are positively associated with knowledge sharing donation behaviors among faculty.

#### Reputation and knowledge sharing collection

Social reputation in the faculty’s field, organization and among peers determine a portion of his/her knowledge sharing collection behaviors [52]. The more reputable a faculty member is, the more likely he or she is likely to perform behaviors aiming at maintaining or boosting that reputation [59]. This includes the creation and dissemination of knowledge [3]. In addition, social reputation makes faculty attempt to attain higher ranks on the prestige or status ladder of his/her field prompting knowledge seeking and collection behaviors [60]. Since the faculty is likely to demonstrate behaviors to protect his/her reputation at all levels, he or she is more likely to collect knowledge that helps in the substance, advancement, and refinement of such reputation [52]. From this logic arises the following hypothesis.

H_9_: Faculty’s reputation is positively associated with knowledge sharing collection behaviors among faculty.

#### Reputation and knowledge sharing donation

Social reputation explains part of why faculty donate knowledge with their peers, networks, and groups. Faculty are more likely to donate knowledge, whether tacit or explicit, to be viewed as credible and reputable by others [38]. In addition, faculty are more likely to donate knowledge to demonstrate how their experience, skills, abilities, and knowledge are worthy of a high reputation rank within different settings [61]. To establish a new reputation within a new team or group, faculty are more likely to donate knowledge to demonstrate their skills’ level, as well as professional affiliations to gain the respect of existing or new members of the network [32]. Faculty also donate knowledge to prove to themselves that they are deserving of the reputation attached to them by others, a form of self-assurance [32]. From such a discussion, one may posit the following hypothesis.

H_10_: Faculty’s reputation is positively associated with knowledge sharing donation behaviors among faculty.

#### Altruism and knowledge sharing collection

Altruism determines a portion in the variance in faculty’s knowledge sharing collection behaviors [33]. To seem amicable, tolerant and of good moral character, faculty attempt to please their colleagues by performing actions accepted as altruistic such as collecting knowledge and sharing it with others [33]. Further, faculty are more likely to collect knowledge to help a dear colleague or a struggling new academic or member in a group [62]. Faculty also may collect new knowledge and share it with others to strengthen the norm of reciprocity in a group or the department. Faculty are more likely to collect knowledge to help their communities or groups they identify with survive, appear in appealing form or boost their reputation donating their time and effort in collecting existing knowledge or creating new ones [40, 63]. Arising from this discussion, one may propose the following hypothesis.

H_11_: Altruism is positively associated with knowledge sharing collection behaviors among faculty.

#### Altruism and knowledge sharing donation

Altruism helps in explaining why many faculties choose to donate knowledge with their peers and networks. Faculty who have a positive predisposition towards altruism desire to assist others in sharing their own explicit and tacit knowledge with them [32]. Further, faculty have a tendency to mentor their students or new colleagues, and therefore attempt to assist them when observing them going through struggles. This help maybe in the form of donating ones’ time, effort, or expertise in creating and sharing knowledge [38, 63]. Faculty also have vested interest in the maintenance of their organizations, teams or networks, and therefore are willing to exert efforts beyond prescribed procedures or contracts and help such institutions by providing them with new or existing knowledge [38]. Accordingly, the following hypothesis applies.

H_12_: Altruism is positively associated with knowledge sharing donation behaviors among faculty

Altruism enhances faculty’s likelihood of donating knowledge sharing [64]. Altruistic faculty feel the need to help others in whatever they need, or anything perceived to be ancillary to their careers or lives, therefore attempt to share important information with them [33]. Further, altruistic faculty feel an obligation for making their organization survive and succeed in accomplishing its stated mission and vision, thus they attempt to supply any useful information to realize such ends [34]. Faculty with low altruistic qualities are more likely to withhold information since their sense of voluntary assistance to others or their organization is low [34, 65]. This tendency may be explained by low trust in others or personal valuation to organizational commitment or loyalty.

All the factors previously described in this theoretical framework were illustrated as a research model in Fig 2.

## Methodology

### Materials and methods

King Saud University’s Research Ethics Committee approved this research with the reference number of KSU-HE-18-235.

### Instrument development

A questionnaire was developed utilizing validated scales from the extant literature on knowledge sharing and social cognitive theory. Minor modifications to some items have been introduced to fit the research context of the study. A panel of 5 experienced faculty specializing in knowledge sharing from Saudi Arabia examined the readability, consistency, suitability and face validity of all items on the instrument. Few minor modifications on the language and wording were suggested and incorporated into the questionnaire. In addition, a pilot study with a sample of 30 Saudi graduate students was performed and their comments also were incorporated to enhance the readability and brevity of some items on the instrument. Table 1 demonstrates the number of items per variable and its source. Note that all items were measured on a Likert-item basis ranging from 1 = Strongly Disagree and 5 = Strongly Agree.

**Table 1:** Instrument development.

Construct	Definition		Items	Reference of Items
Self-efficacy	People’s judgments of their capabilities to organize and execute courses of action required to attain designated types of performances. It is concerned not with the skills one has, but with the judgments of what one can do with whatever skills one possesses.		1. When I share knowledge with others, I will gain more respect and recognition. 2. When I share knowledge, I will have more friends. 3. When I share knowledge, people will regard me as a dependable person. 4. When I share knowledge, I will have better ties between me and others. 5. When I share knowledge, I will get benefits. 6. When I share knowledge, I will be happy. 7. When I share knowledge, I will feel myself as a successful person.	Bandura [66]
Personal outcome expectations	The personal benefits that are generated by specific personal behaviors, including personal rewards and promotions.		1. Sharing knowledge with others makes me feel smarter. 2. Sharing knowledge with others makes me feel satisfied. 3. I am capable of authoring scholarly works and posting threads in discussion forums. 4. Sharing knowledge with others because I might lose my job otherwise. 5. Sharing knowledge with others because I might be punished otherwise. 6. Sharing knowledge with others is interesting because I can see that I can influence people. 7. I allocate time for sharing knowledge with others.	Wang [67]
Community-related outcome expectations	An individual’s expectations about the impact of knowledge sharing on… achieving the goals, enriching knowledge base.	1. I help my community positively when I engage in knowledge sharing. 2. My knowledge sharing helps my community to keep its status among other communities. 3. With my knowledge sharing, my community continue to function. 4. With my knowledge sharing, I can help to promote cumulative knowledge in my community. 5. With my knowledge sharing, I can help to the growth of community.		Hsu et al. [49]
Reputation	An important asset that an individual can leverage to achieve and maintain status within a collective.	1. Knowledge sharing helps me to gain respect. 2. Knowledge sharing enhances my status. 3. Knowledge sharing improves my reputation. 4. Knowledge sharing lets me get feedback on my status and reputation.		Wasko & Faraj [68]
Altruism	Voluntary actions or enjoyment to help other participants in a virtual community.	1. Altruism due to participation is positively related to the quality of knowledge sharing. 2. Altruism due to participation is positively related to the quantity of knowledge sharing. 3. When someone has a higher participation level, altruism due to participation is positively related to the quality of knowledge sharing. 4. When someone has a higher participation level, altruism due to participation is positively related to the quantity of knowledge sharing.		Chang & Chuang, [69]
Knowledge sharing (Donating)	Communicating to others what one’s personal intellectual capital is.	1. When I learn something new, I share it with my department. 2. I share knowledge with people at my department 3. I share my skills with people at my department 4. When I learn something new, I share it with people outside of my department. 5. I share knowledge with people outside of my department		Van den Hoof & de Ridder [70]
Knowledge sharing (Collecting)	Consulting colleagues in order to get them to share their intellectual capital.	1. When I ask a question to my colleague at my department, he/she answers it. 2. When I want to learn about their skills, my colleagues mention their skills. 3. When I ask a question to my colleague outside of my department, he/she answers it.		Van den Hoof & de Ridder [70]

### Sample

The population of the study is all Saudi faculty teaching at higher learning institutions. Constructing a sampling frame to be used for the creation of a random-probability sample is extremely difficult and time-consuming. An email invitation was sent to all teaching and research faculty working at the researcher’s institution. 411 completed questionnaires were used as the sample for this research. Table 2 demonstrates the distribution of participants based on selected demographic variables. Notice that this is the first study performed in Saudi Arabia on KS activities featuring more females compared to males in faculty.

**Table 2:** Demographics analysis of Saudi faculty and social media.

Demographics Analysis		
		Percent
Age	Average = 35 years	
Gender	Female	51.6%
	Male	48.4%
Education	Master	34.3%
	Ph.D.	65.7%
Major	Art	10.4%
	Computer	14.9%
	Education	14.3%
	Engineering	10.7%
	Management	12%
	Medical	10.5%
	Science	22.7%
	Other	4.2%
Have at least one account on social media	Yes	99.4%
Have more than one account on social media	Yes	93.5%
Years of using social media	less than 1 year	0.5%
	1–3 years	2.0%
	3–5 years	5.2%
	5 years or more	92.3%
I am a member of	Facebook	61%
	Twitter	92%
	LinkedIn	59.7%
	WhatsApp	99%
	Snapchat	88.2%
	Other	67.2%

Note that Table 2 presents information on Saudi faculty’s social media use. This is important since the bulk of knowledge sharing activity reported by Saudi instructional and research staff in the literature take place on their personal social media pages. It has also been found that the more a faculty member utilizes social media, and the higher number of accounts he or she possesses, the more likely they engage in knowledge sharing on their personal pages. The inclusion of social media items on the questionnaire was only used for informational purposes to better assess the prevalence of social media use among faculty for knowledge sharing purposes. The email invitation to participate in the research project was sent by the institutional research office directly to the designated emails of participating faculty, and no analysis of knowledge sharing activities on social media was performed for the current manuscript.

### Survey administration

The institutional research office at the author’s university distributed the questionnaire electronically to all full-time teaching and research academics. The questionnaire was embedded into a Google Forms page with an electronic consent form that was necessary to be signed by agreeing to the terms and conditions of voluntary participation in the research. The email was sent twice for all faculty. 524 questionnaires were returned out of which 411 were complete. A total of 732 requests to participate were sent yielding a response rate of 56%.

### Data analysis

Structural equation modelling was used to test the measurement and structural models in Fig 1. AMOS 22 was used for performing the analysis. First, confirmatory factor analysis (CFA) was used to assess the validity of the measurement model. Second, internal consistency analysis was conducted to evaluate the reliability of the instrument. Third, estimation of the relationships was performed using maximum likelihood procedures built in the software to determine the strength and direction of the associations linking hypothesized independent variables with the dependent variable.

### Instrument validation

Table 3 demonstrates the results of the CFA indicating high construct validity for all constructs included in the study. Notice that all loadings are above 0.5 and all constructs were permitted to co-vary while each item was modelled as a reflective indicator of its construct. The table also alludes to the fact that most of the sample agreed with the statements presented to them highlighted by relatively high means.

**Table 3:** CFA results.

Item	Mean	Loading	Item	Mean	Loading
SEF1	4.48	0.83	COE5	3.92	0.57
SEF2	4.28	0.77	REP1	4.56	0.74
SEF3	4.18	0.72	REP2	3.83	0.69
SEF4	3.87	0.68	REP3	4.21	0.64
SEF5	4.33	0.63	REP4	4.7	0.61
SEF6	3.82	0.55	ALT1	4.11	0.76
SEF7	3.92	0.52	ALT2	4.31	0.73
POE1	4.47	0.81	ALT3	4.65	0.63
POE2	4.42	0.78	ALT4	3.54	0.58
POE3	4.7	0.75	KD1	4.42	0.69
POE4	4.5	0.71	KD2	4.70	0.67
POE5	4.19	0.67	KD 3	4.50	0.61
POE6	4.71	0.59	KD 4	3.92	0.59
POE7	4.49	0.53	KD 5	4.26	0.54
COE1	3.76	0.75	KC1	4.30	0.73
COE2	4.22	0.72	KC2	4.33	0.64
COE3	4.37	0.67	KC3	4.70	0.61
COE4	3.68	0.63			

Table 4 displays the inter-item correlations among all the constructs, their average explained variance (AVE) and squared rooted root of average explained variance’s value. Convergent validity of the constructs is assessed by looking at factor loadings and average explained variances. Note that average explained variances are all above 0.50, exceeding measurement error, and all loadings are above 0.5 indicating consistent internal robust structure, which are signs of convergent validity [71]. Discriminant validity is assessed by looking at the inter-item correlations among all variables and the square root of average explained variance. Note that all inter-item correlations among variables are below 0.5 noting to the independence of each construct and the square root of average explained variance is larger than bivariate correlations suggesting discriminant validity for all constructs [72].

**Table 4:** Inter-item correlations.

	AVE	SEF	POE	COE	REP	ALT	KD	KC
SEF	0.62	**0.78**						
POE	0.66	0.48	**0.81**					
COE	0.68	0.42	0.44	**0.82**				
REP	0.64	0.55	0.32	0.29	**0.80**			
ALT	0.63	0.16	0.34	0.10	0.45	**0.79**		
KD	0.62	0.37	0.24	0.30	0.23	0.35	**0.78**	
KC	0.68	0.48	0.29	0.46	0.21	0.26	0.34	**0.82**

Each diagonal value shown in bold represents the square root of its construct’s average explained variance value. The values under each diagonal value represent the correlations among the related constructs. Each diagonal value was found to be greater than the correlations among the related constructs.

Table 5 displays Cronbach alphas and composite reliabilities for all constructs included in the questionnaire. Note that all alphas are above 0.70 indicating the suitability of scales for research use [71]. All constructs have composite reliabilities of above 0.70 indicating that the construct reliability and internal consistency are high [71]. Not shown for brevity, item-total correlations for all items were above 0.5 indicating that all items are consistent and stable [73].

**Table 5:** Cronbach’s Alpha and composite reliability scale.

Scale	Cronbach’s Alpha	Composite Reliability
Self-Efficacy (Slf_EF)	0.74	0.84
Personal Outcome Experience (POE)	0.79	0.87
Community-related Outcome Experience (COE)	0.79	0.89
Reputation (REP)	0.74	0.87
Altruism (ALT)	0.73	0.85
Knowledge Donating (KD)	0.78	0.89
Knowledge Collecting (KC)	0.76	0.88

## Results

The model presented in Fig 2 was tested using the original data obtained from Saudi faculty. Table 6 displays the goodness of fit indices for the structural model. The statistics obtained fall within recommended guidelines. The χ2 to degrees of freedom ratio of 3.45 (χ2 = 2383; df = 972), IFI = 0.88, and RMSEA = 0.064 [71, 74, 75]. The measurement model demonstrated acceptable validity and reliability as was shown in the instrument validation section.

**Table 6:** Goodness of fit indices.

Fit Index	Results	Recommended Criteria	Authors
CMIN/DF (χ^2^/DF)	2383/690 = 3.45	≤ 5	Hair et al. [71]
RMSEA	0.064	≥ 0.06	Hu and Bentler [74]
IFI	0.881	≥ 0.80	Hu & Bentler [74]
NNFI	0.972	≥ 0.90	Bentler & Bonett [75]

**Fig 3:**
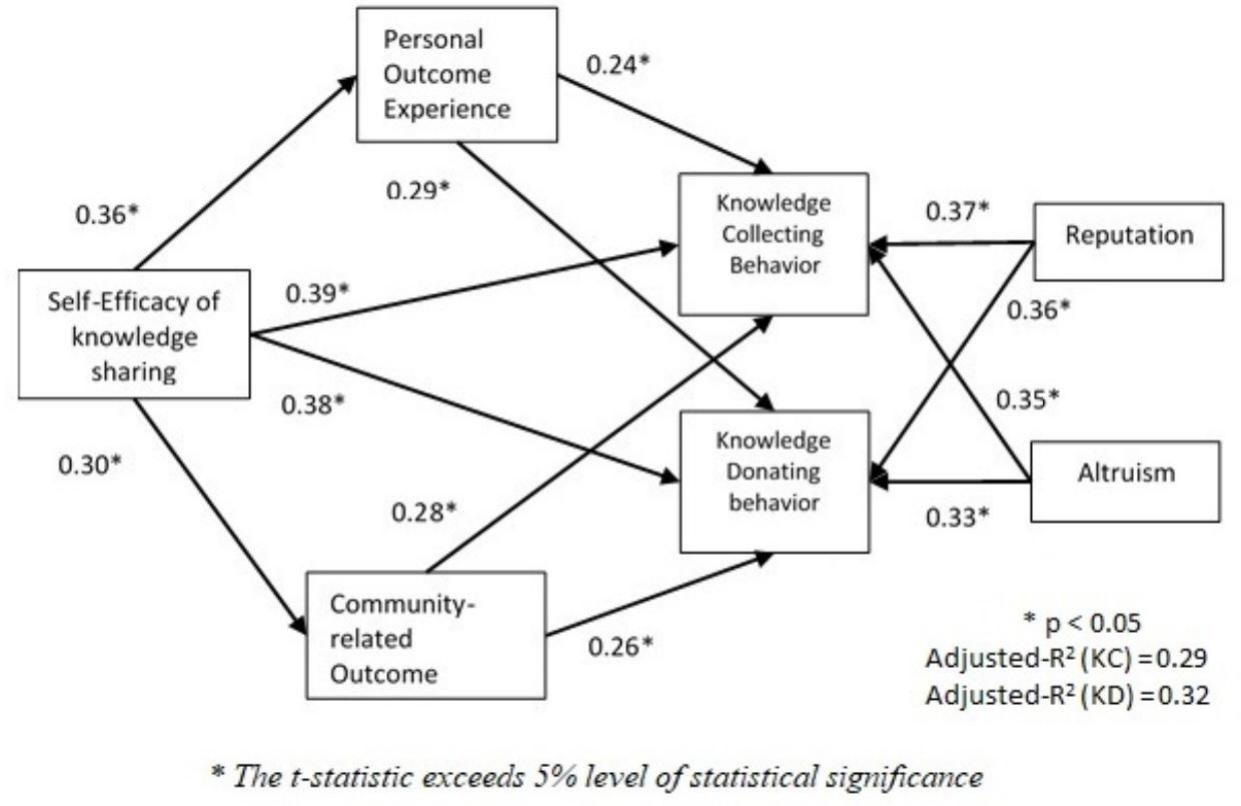
Structural model.

Fig 3 and Table 7 demonstrates the estimates of the SEM structural model showing path coefficients linking independent variables to the dependent variable. All twelve hypotheses were supported, and coefficients were significant at the 0.05 significance. Both personal and community expectations outcomes were found to be significant predictors of faculty’s knowledge sharing collection and donation. Self-efficacy of knowledge sharing is the strongest predictor of knowledge sharing collection and donation since it has direct and indirect effects on knowledge sharing activities. Community expectations, however, seem to have a stronger effect on knowledge sharing collection and donation compared to personal expectations.

**Table 7:** Path coefficients and their significance.

Hypothesis	Path	Coefficient	T-Values	Supported or not
H_1_	Self-Efficacy→ Personal Expectations Outcomes	0.36*	6.41	Yes
H_2_	Self-Efficacy→ Community Related Outcomes	0.30*	6.65	Yes
H_3_	Self-Efficacy→ Knowledge Collecting Behaviors	0.39*	5.60	Yes
H_4_	Self-efficacy→ Knowledge Donating Behaviors	0.38*	3.65	Yes
H_5_	Personal Expectation Outcomes→ Knowledge Collection Behaviors	0. 24*	7.03	Yes
H_6_	Personal Expectation Outcomes→ Knowledge Donation Behaviors	0.29*	3.19	Yes
H_7_	Community Related Outcomes→ Knowledge Collection Behaviors	0.26*	5.23	Yes
H_8_	Community Related Outcomes→ Knowledge Donation Behaviors	0.28*	5.79	Yes
H_9_	Reputation→ Knowledge Collection Behaviors	0.37*	4.27	Yes
H_10_	Reputation→ Knowledge Donation Behaviors	0.36*	3.68	Yes
H_11_	Altruism→ Knowledge Collection Behaviors	0.35*	5.38	Yes
H_12_	Altruism→ Knowledge Donation Behaviors	0.33*	7.25	Yes

* p<0.05

Adjusted R-Squared for Knowledge Collection Behaviors is 0.29

Adjusted R-Squared for Knowledge Donation Behaviors is 0.32

Personal expectations carry the weakest effects on knowledge sharing collection and donation (0.24 and 0.29). Personal community-related expectations also have weaker effects on knowledge sharing collection and donation compared to actual community-related outcomes, reputation, and altruism. Reputation is a stronger predictor of knowledge sharing collection and donation compared to altruism. Knowledge sharing self-efficacy is a significant predictor of personal expectations of knowledge sharing. In addition, knowledge sharing self-efficacy is the best predictor among all included for knowledge sharing collection and donation among Saudi faculty.

The explanatory power of the model is indicated by the adjusted R-squared of 0.29 and 0.32 for knowledge collection and donation respectively. This indicates that twenty nine percent in the variation of Saudi faculty knowledge sharing collection is explained by social cognitive theory. By the same token, thirty two percent of the variance in the knowledge donation behaviors among Saudi faculty is explained by social cognitive theory. Note that the explanatory power of all included constructs has similar magnitude, coefficients range between 0.24 and 0.37, which is not a very large difference given that the data used is based on Likert-based data ranging from 1 to 5 for each item.

## Discussion and conclusions

The findings of this research are consistent with previous evidence supporting the relevance of social cognitive theory as a potent explanatory framework for knowledge sharing behaviors. More specifically, the research shows the strong predictive weight of self-efficacy and community characteristics like reputation and altruism in academic circles in influencing knowledge sharing proclivities. The research also confirms the relevance of how individual attitudes concerning the tangible benefits one obtains from knowledge sharing, as well as the positive gains groups one identifies with acquiring from knowledge sharing make individuals more likely to practice knowledge sharing activities.

The findings of study provide a blueprint to remedy the gaps in knowledge sharing performance by Saudi faculty, and higher learning institutions. First, the results alluded to the importance of community related outcomes including what others in the institution perceived the work of an individual faculty or unit to be worth [36, 49]. This indicates that linking rewards, recognition, and promotion to knowledge sharing activities is more likely going to enhance the activity at levels. More importantly, setting a clear vision, an implementation plan, and an evaluation tool for knowledge sharing, and applying it consistently and regularly, on all personnel and administrative divisions would likely increase the rate at which Saudi faculty and institutions practice KS.

The results of the analysis aids stakeholders, educators, and policymakers in removing the barriers preventing Saudi faculty from exercising KS. First, the relative power of self-efficacy in predicting KS activities suggest a further need in the investment of professional development to demonstrate exemplary KS endeavors for new, as well as senior faculty and administrators. Second, the importance of altruism in predicting the collection and donation behaviors of knowledge sharing presents an opportunity for colleges and universities to mandate each department, college, or division to set up formal channels of knowledge sharing on their sites, social media pages and electronic databases. The investment in the formal organizational infrastructure in knowledge sharing is likely to send a leadership commitment message to faculty motivating them to further apply the recommended behaviors especially when it is rewarded, easy to implement, and recognized [12].

This research contributes to the practice and theory of knowledge sharing in higher education in many ways. First, this research highlights the need for the development of validated professional training programs on knowledge sharing for faculty in Saudi Arabia. Such programs should be tailored to enhance faculty’s knowledge sharing self-efficacy, their skills in translating tacit knowledge into explicit forms of knowledge and how to appropriately share it on social media platforms. Second, the findings allude to the need for departments, colleges, and offices across Saudi universities for creating official well-supported knowledge sharing platforms using formal channels on social media. Within such platforms, the institution should link knowledge sharing activities with promotion criteria through the installation of mentorship programs where senior faculty train new hired academics. Third, institutions need to incentivize knowledge sharing by providing small grants for faculty to share their tacit knowledge on official channels/platforms on social media where more faculty benefit from this [18]. On the theoretical level, this paper demonstrated the validity of social cognitive theory in explaining knowledge sharing in a new context, Saudi faculty. In addition, the scales utilized in the questionnaire have been validated in the same novel setting. This paves the way for future research relying on the theory and measurement tools deployed throughout the current manuscript.

The findings allude to the importance of organizational rewards, incentives, and recognition linked to knowledge sharing activities in higher education. Reputation matters in academia. Once universities, colleges, chairs and deans reward faculty members for their efforts in disseminating specialized teaching, research, and service knowledge with their peers and administrators, faculty are more likely to adopt collection and donation behaviors. Recognizing exemplary knowledge sharing endeavors facilitates its growth on campus. Saudi universities and colleges do not engage in sufficient rewarding nor recognition resulting in diminished knowledge sharing activity.

This research highlights the importance of a previously neglected variable in higher education knowledge sharing research, altruism. Saudi universities and colleges have thus far failed to establish communities of practice, active mediums and channels of knowledge sharing paving the way for altruistic faculty to share their knowledge. On many occasions, faculty attempt to reach out the largest number of beneficiaries from his or her knowledge opting for the use of their personal sites, a finding in the Saudi higher education literature [8, 9]. This is partly due to the absence of lively well-maintained spaces provided by faculty’s institutions. The failure of constructing such forums resulted in stunted growth in the knowledge sharing realm in the Saudi higher education sector.

### Theoretical implications

One of the main findings emerging from the current manuscript is the invariance of social cognitive theory. Using a previously unexamined context with respect to the model, the Saudi faculty, the theory explained a respectable proportion of variance in knowledge sharing behaviors [8, 17, 18]. This indicates the stability and relevance of the social cognitive theory in explaining the incidence and maintenance of important behaviors outlined by its original developer Bandura [24, 25]. Further, the original data collected from the sample provided further validation evidence to the utilized questionnaires from the extant literature as outlined in Table 1.

Social cognitive theory is susceptible to change based on the research context, and a modification of the model is necessary to account for the variance in the outcome across many settings [67]. For studying knowledge sharing in higher education, this study provided support for the roles of reputation and altruism in directly explaining faculty’s knowledge sharing [59, 60]. This suggests the need of including the constructs in future models aimed at predicting knowledge sharing in educational environments. The current research also presented evidence to the reliability and validity of the questionnaires measuring both constructs.

This research confirms the central role of behavioral capability in social cognitive theory. One of the strongest predictors found to improve faculty’s knowledge sharing collection and donation is self-efficacy [29]. A faculty’s belief in his or her ability to collect and donate knowledge is crucial in the amount and quality of knowledge to be disseminated to peers or on platforms. This indicates that any instrument measuring social cognitive theory lacking an explicit assessment of self-efficacy or behavioral capacity is inadequate [12, 35, 49].

The use of social cognitive theory in the social, management or behavioral sciences must be linked to the context of research under consideration. This study introduced the role of altruism and reputation as major drivers of faculty behaviors in academia. While the constructs may not extend to other contexts, their absence of knowledge sharing research among academics, students or educational settings will likely generate inaccurate estimates. Both constructs were found to be significantly related to knowledge sharing in academic settings, and their inclusion in theoretical models in similar contexts is a necessity.

### Implications for practice

This research highlighted the importance of self-efficacy related to knowledge sharing in facilitating knowledge sharing activities. This indicates the importance in providing faculty professional development courses/modules on the values of knowledge sharing; how to communicate implicit knowledge and share explicit knowledge with others on social media platforms. This finding is consistent with previous analyses of Saudi faculty knowledge sharing behaviors. The extent literature has concluded a low to medium appreciation of knowledge sharing among Saudi faculty. Authors have recommended the construction of specialized workshops to new faculty in order to enhance their knowledge sharing recognition and practice [8, 17, 18]. This effort is further improved by the establishment of communities of practice interested in knowledge sharing at the college or departmental level. This will enhance the personal career development of faculty, as well as allow senior members to be better recognized and boost their reputation. Also, the founding of faculty mentoring initiatives at the departmental level and shifting them virtually where more faculty across the university or college benefit from the information would increase faculty’s self-efficacy and practice of knowledge sharing.

Saudi higher education institutions need to materialize on the pervasive social media use exhibited by their faculty, and their altruistic tendency of assisting each other improve personal knowledge, skills, and abilities, as well as their work units. Previous research in Saudi Arabia has shown a high utilization rate of Twitter among Saudi faculty to transmit explicit and implicit work-related knowledge with their peers. Additionally, higher education analysts in Saudi Arabia have pointed out to the rise of creative initiatives pioneered by enthusiastic and talented faculty to transform teaching research and service in the sector [5, 9, 10]. This can be captured through the sponsoring of formal channels facilitating knowledge sharing activities on social media platforms. Departments, colleges and offices throughout the organization should launch their virtual communities of practice and incentivize faculty’s participation. The inclusion of knowledge sharing into promotion criteria as a standard of service to the department, college and the university will guarantee higher engagement of faculty in knowledge sharing activities through the virtual channels. Such channels will be an official business item on departmental, college and office-related meetings’ agendas and a culture of knowledge sharing is inculcated in such settings by top leadership.

### Limitations

This research is based on survey data, which is sensitive to many biases including social desirability and agreement acquiescence. Further, convenience sampling presents a problem for generalizability. The use of a single large organization may not account for systematic differences across academic settings in Saudi Arabia. Such findings presented above would not extend beyond the Saudi context giving the differences in academic culture, social expectations and settings governing the behaviors of faculty across countries.

### Future research

Future research should utilize non-survey data for testing the predictors of knowledge sharing activities. This includes measures such as the frequency of posting materials on social media groups, the type of information shared on social media pages and the quality of such information. For example, game theoretical analysis can be a useful approach to show the interaction between various players [76, 77]. Especially, this method can help to illustrate the tendency for information exchange between faculty in higher education [78]. In this way, future researchers can also account for knowledge sharing strategies in higher education [79]. In addition, experimental methods should be more utilized in the investigation of knowledge sharing to guide the development of interventions capable of enhancing knowledge sharing activities. Controlled and natural experimental methods are widely available for researchers to investigate individuals’ knowledge sharing behaviors. This adds more reliability, validity, precision and accuracy of the findings.

Future researchers should concentrate on the context of the study and develop suitable measures using relevant frameworks. For instance, academic settings differ drastically from industry work environments rendering some variables inappropriate. The use of reputation and altruism in studying knowledge sharing activities in the private or public sector are not the most useful community-related characteristics. Every setting is unique and requires the researcher to develop relevant variables with validated metrics to better understand knowledge sharing behaviors.

## Conclusions

This investigation sheds light on the inadequate knowledge sharing activity among Saudi faculty. The present analysis has shown the significance of increasing faculty’s self-efficacy, personal expectations, and community outcomes perceptions on the improvement of knowledge sharing behaviors. Results have supported proposals for investing in faculty development programs aiming for the enhancement of knowledge sharing in higher education. Simultaneously, findings have demonstrated the relevance of social cognitive interventions in resolving the low implicit knowledge sharing trends among Saudi faculty.

This empirical study featured a sample of 411 Saudi faculty at a large public university. The authors developed a structural equations model consisting of 12 hypotheses and 7 multi-items constructs. Results have supported all hypotheses and demonstrated reliability and validity of the questionnaire utilized. Social cognitive theory has explained approximately 30% in knowledge sharing activity among Saudi faculty. The investigation has shown the need to improve faculty’s recognition and altruism within the workplace to generate higher levels of knowledge sharing.

Saudi colleges and universities are encouraged to collect regular data on knowledge sharing active across their organizations. Non-survey and longitudinal data projects are needed to establish better interventions enhancing knowledge sharing in higher education. The use of social media platforms should be integrated into professional development modules delivered to Saudi faculty for knowledge sharing improvement purposes. Top management support for knowledge sharing is a requisite ingredient for achieving the optimal level of knowledge sharing.

## Supporting information

S1 Data (XLSX)
